# Pelvic Organ Prolapse: The Regenerative Medicine Approach

**DOI:** 10.1089/gyn.2019.0042

**Published:** 2019-12-09

**Authors:** Magdalene Karon

**Affiliations:** Department of Pelvic Reconstructive Surgery, CHI St. Joseph Health, Lexington, KY.

Editor: On April 16, 2019, the U.S. Food and Drug Administration (FDA) issued a statement for immediate release in a press release entitled: “FDA Takes Action to Protect Women's Health, Orders Manufacturers of Surgical Mesh Intended for Transvaginal Repair of Pelvic Organ Prolapse to Stop Selling All Devices.”^1^

This journal recently featured a well-published article on transvaginal pelvic organ prolapse mesh repair.^2^ However, in light of new FDA warnings, these techniques will no longer be applicable for patients who need surgical correction of their pelvic organ prolapses. Both surgeons and patients will be looking for other options.

Before these meshes were introduced by industry, our residency training programs taught us the pertinent anatomy and native tissue repairs both transvaginally and transabdominally. Such classic operations as sacrocolpopexy, paravaginal defect repair, and Burch colposuspension were part of our training and still remain timeless.

I had the privilege of knowing David Nichols, MD, one of the greatest vaginal surgeons, and was lucky to have had great mentors during my training. Currently, more often than necessary, we are taught by a salesperson from industry how to implant a device or use a product that profits that company over its competition even when there is questionable benefit for patients. Perhaps it is time to revisit our classic operations that can now be performed laparoscopically rather than by open laparotomy.

Perhaps it is time to recall those lessons in anatomy and surgical techniques, and now armed with better suture materials and optics, to consider replacing synthetic mesh with biologic matrix patches. Mesh implants work on the principle of stimulating a foreign-body reaction and fibrosis resulting in a dense avascular scar that is not viable tissue. Conversely, biologic grafts function on the principle of a “scaffold,” which promotes tissue regeneration by attracting stem cells and macrophages to the targeted area, specifically decellularized noncrosslinked grafts. This approach harnesses and amplifies the endogenous healing potential to fill in the tissue defect by mimicking the tissue type next to which it is placed.

Interestingly, the same manufacturers of the synthetic meshes are starting to study the market of decellularized matrix grafts. With caution, we might need to start exploring these options. I have published the first study in 9 patients in this journal, using such mesh alternatives in sacrocolpopexy.^3^ We have now expanded this research to 211 patients with data under review.

I, too, was taught the “scar makes it strong” concept. Regenerative healing is a different concept, but it is a better one, because it promotes new viable and vascularized tissue rather than a dense scar and adhesions.

There is still a long way to go to change our thoughts on this paradigm. However, I say let's move on to the newer approach to regenerative healing.
